# A case and video presentation using rotational ETOSS of intentional route tracing by angiography-based 3D wiring in CTO-PCI

**DOI:** 10.1007/s12928-022-00861-3

**Published:** 2022-05-03

**Authors:** Atsunori Okamura, Hiroyuki Nagai, Kota Tanaka, Satoshi Suzuki, Heitaro Watanabe, Katsuomi Iwakura

**Affiliations:** Division of Cardiology, Cardiovascular Center, Sakurabashi Watanabe Hospital, 2-4-32 Umeda, Kita-ku, Osaka, 530-0001 Japan

**Keywords:** Coronary intervention, Chronic total occlusion, 3D wiring

In chronic total occlusion (CTO) intervention, the difference between the angiography-based 2-dimensional (2D) and 3-dimensional (3D) wiring has not been recognized worldwide. Candidate lesions for 3D wiring are lesions in which the target can be visually recognized within 1 cm by contrast medium or calcification. 3D wiring is the method of tracing the ideal route imaged from virtual or visualized vessel walls while recognizing the rotational direction of a guidewire using the 3D imaging rule (3D-R) to converge the tip to the final target. The advantages of it are to increase the success rate of antegrade wiring and reduce the risk of guidewire perforation [1]. We report a case of accurately tracing the central route of the CTO lesion imaged from calcified vessel walls using 3D-R and show video demonstrations using Rotational ETOSS (Asahi Intecc Co., Ltd., Aichi, Japan) to clearly show the difference between 2 and 3D wiring.

A 67-year-old man suffered from angina pectoris due to the CTO in the left circumflex coronary artery (Fig. 1A). The CTO lesion was visualized by calcifications around the vessel wall. A XT-R (Asahi Intecc Co.) could not enter the CTO, therefore we advanced a Confianza 12 g with a 1.0-mm curve at an angle of 45° supported by a Corsair (Asahi Intecc Co.). By the fluorographic observation from two orthogonal directions without contrast medium (Fig. 1–1, 2), the rotational direction of the guidewire was determined using 3D-R and the guidewire was rotated clockwise to direct the tip toward the center of the vessel and then advanced (Fig. 1–3). The same procedures were repeated as shown in Fig. 1–3–13. The guidewire motion became free at the exit (Fig. 1–14) and coronary angiography showed that the guidewire was inside the distal lumen (Fig. 1–15). Normal flow was achieved after stent implantation (Fig. 1B).

**Fig. 1:**
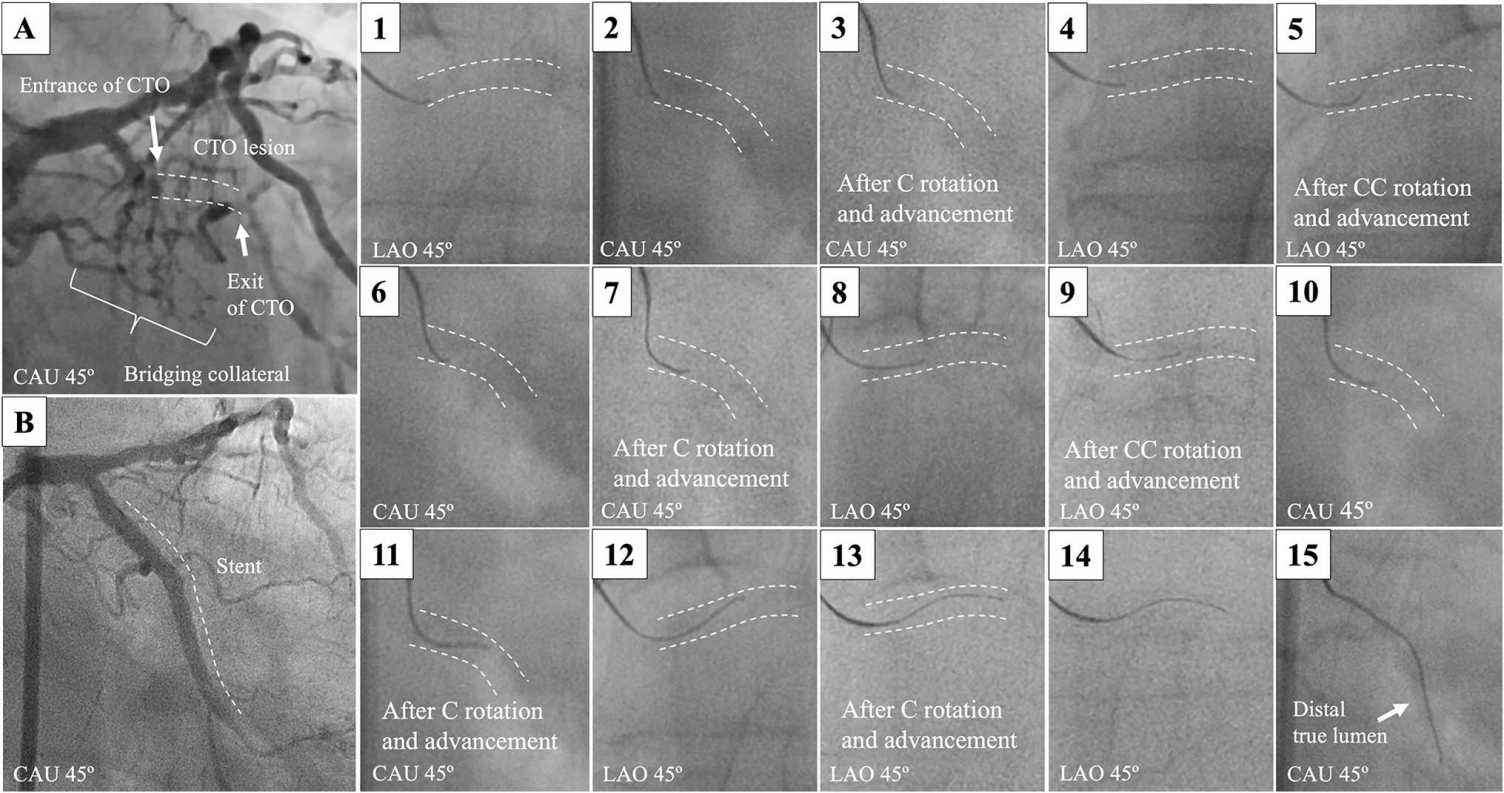
Angiographic images **A** prior to and **B** after the procedure. (1–14) Fluorographic images and (15) angiographic image during 3-dimensional wiring. The dotted lines show the vessel walls. *CAU* caudal, *C* clockwise, *CC* counterclockwise, *CTO* chronic total occlusion, *LAO* left anterior oblique

## Supplementary Information

Below is the link to the electronic supplementary material.

Supplementary file1 Video 1.2D wiring-1: In one observation direction, the apex of the tip was directed toward the target without paying attention to the rotational direction of the guidewire and then advanced. (MP4 24545 KB)

Supplementary file2 Video 2.2D wiring-2: The observations were repeated in two directions until the apex of the tip was directed toward the targets and then advanced. (MP4 35729 KB)

Supplementary file3 Video 3.3D wiring: In one observation direction, the rotational direction of the guidewire was determined using the 3D imaging rule, and the apex of the tip was directed toward the target and then advanced. (MP4 19831 KB)

## Abbreviations

2D: 2-Dimensional
3D: 3-Dimensional
3D-R: 3-Dimensional imaging rule
CTO: Chronic total occlusion
